# Epigenetic Modulation and Brain Function in Parkinson's Disease: The Role of Exercise Alone or Combined With Polyphenols

**DOI:** 10.1002/fsn3.70696

**Published:** 2025-07-28

**Authors:** Xuenan Cao, Xiantao Cui, Bita Badehnoosh

**Affiliations:** ^1^ Shanghai Zhongqiao Vocational and Technical University Shanghai China; ^2^ Shanghai Jinshan District Mental Health Center Shanghai China; ^3^ Department of Gynecology and Obstetrics, Dietary Supplements and Probiotic Research Center Alborz University of Medical Sciences Karaj Iran

**Keywords:** epigenetic changes, exercise, microRNA, Parkinsons disease, polyphenols

## Abstract

Parkinson's disease (PD) is a complex, progressive neurodegenerative disorder that affects millions worldwide and is a growing contributor to disability. While pharmacological treatments remain central to symptom management, they often fall short in altering disease progression. Increasing evidence points to epigenetic modifications, such as DNA methylation, histone acetylation, and noncoding RNA regulation, as crucial factors in PD pathogenesis and promising targets for intervention. These molecular mechanisms govern gene expression without changing the DNA sequence and are influenced by environmental and lifestyle factors. Among these, physical exercise and dietary polyphenols have emerged as potent, noninvasive modulators of the epigenome. Exercise has been shown to alter DNA methylation, increase histone acetylation, and regulate microRNAs and long noncoding RNAs in ways that support neuronal resilience. Similarly, polyphenols affect epigenetic marks and reduce oxidative stress and inflammation, both key drivers of PD pathology. Therefore, in this review, we explore how these interventions, individually and in combination, influence epigenetic mechanisms relevant to PD. We summarize current evidence on the neuroprotective and regulatory effects of exercise, and highlight studies investigating the potential synergy of exercise and polyphenols. By synthesizing these findings, our goal is to provide insight into how these strategies may contribute to personalized, epigenetically informed approaches for managing and potentially modifying the course of PD.

## Graphical abstract

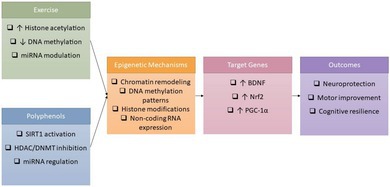

## 1 Introduction

Parkinson's disease (PD) is currently the second most prevalent neurodegenerative disorder worldwide, affecting over six million people, a number that has grown dramatically, more than doubling in the past two decades (Feigin et al. 2019; Dorsey et al. 2018). This sharp rise places PD among the top causes of neurological disability. The disease is marked by the presence of Lewy bodies and Lewy neurites, especially in the substantia nigra, which leads to the progressive loss of dopamine‐producing neurons. These inclusions mainly consist of misfolded and aggregated α‐synuclein, categorizing PD as a synucleinopathy. While the Braak staging model outlines a potential pathway of Lewy pathology spreading from the lower brainstem to the cortex, the progression can vary significantly between individuals (Braak et al. 2002). Recent findings support the theory that PD pathology may propagate through a prion‐like mechanism involving the cell‐to‐cell transmission of abnormal α‐synuclein (Steiner et al. 2018). Although PD is strongly associated with aging, it does not solely affect the elderly people. Approximately one‐quarter of cases develop before the age of 65, and up to 10% of individuals are diagnosed before turning 50. Cases with onset before 40 are often referred to as young‐onset PD. The disease affects people across all regions, with rapid case growth particularly noted in China and high‐income European nations (Dorsey et al. 2018; Deuschl et al. 2020). Notably, gender differences have also emerged: Although men are more likely to develop PD and suffer longer with disability, women often face greater challenges related to treatment side effects, such as dyskinesia and mood fluctuations (Deuschl et al. 2020; Pringsheim et al. 2014; Bjornestad et al. 2016; Picillo et al. 2016). Additionally, women tend to delay seeking care and are less likely to receive advanced interventions, including surgery (Fullard et al. 2017, 2018). They also remain underrepresented in clinical research (Tosserams et al. 2018).

Traditional treatment begins with dopamine replacement therapies such as levodopa or dopamine agonists (Church 2021). However, due to the complex and variable nature of PD, many patients turn to integrative approaches, including lifestyle modifications. Exercise, particularly aerobic activity, has shown neuroprotective effects and helps manage both motor and non‐motor symptoms. A comprehensive care strategy, combining medication, rehabilitation, physical activity, and when necessary, surgical intervention, offers a more holistic approach. Such a plan addresses PD's multifaceted impact and adapts to the individual needs of each person living with the disease (Church 2021).

Epigenetic alterations, long recognized in cancer, are now understood to play crucial roles in many chronic conditions including diabetes, autoimmune disorders such as lupus, respiratory diseases such as asthma, and a wide array of neurological disorders (Fouse and Costello 2009; Villeneuve and Natarajan 2010; Javierre et al. 2010; Adcock et al. 2005; Urdinguio et al. 2009; Feng and Fan 2009). These modifications include changes in DNA methylation, histone acetylation and methylation, and expression of chromatin‐modifying enzymes, all of which can lead to altered gene expression without modifying the underlying genetic code. In many disease states, global DNA hypomethylation can result in genomic instability, while hypermethylation of gene promoters, especially those located in CpG islands, can lead to the silencing of genes important for normal cellular function. Additionally, loss of specific histone marks such as H4K16 acetylation and H4K20 tri‐methylation, as well as increased expression of repressors such as BMI1 and EZH2, further disrupts normal gene regulation (Jones and Baylin 2007; Sharma et al. 2010). Interestingly, genes that are typically targeted by the polycomb repressive complexes in embryonic stem cells appear more susceptible to methylation in disease conditions, linking early developmental epigenetic patterns to later disease susceptibility (Widschwendter et al. 2007; Schlesinger et al. 2007; Ohm et al. 2007).

Epigenetic signatures are increasingly being investigated for their potential to stratify diseases, predict clinical outcomes, and monitor treatment responses (Shen, Toyota, et al. 2007; Seligson et al. 2005; Figueroa et al. 2010). For instance, distinct histone modifications have been shown to differentiate diseased tissue from healthy controls, and specific methylation patterns can correlate with treatment response and disease recurrence (Ellinger et al. 2010; Bachmann et al. 2006; Kanai 2010). In neurological disorders, such epigenetic marks may be useful in identifying individuals at higher risk, tracking disease progression, or tailoring personalized treatment plans. With the growing advancement of epigenome profiling technologies, the emerging field of pharmaco‐epigenomics offers new possibilities in personalizing therapies for complex diseases beyond cancer (Shen, Kondo, et al. 2007). Epigenetic patterns can reveal critical regulatory pathways involved in drug metabolism and response, guiding more precise treatment strategies. For example, individual differences in DNA methylation or histone modification profiles may help predict drug sensitivity, dosing requirements, or adverse reactions. Additionally, tracking epigenetic changes over time may serve as a useful biomarker to monitor therapeutic efficacy or disease progression (Kelly et al. 2010). Because epigenetic marks are dynamic yet reversible, they offer an appealing target for intervention with drugs designed to restore normal gene expression without altering the DNA sequence (Kelly et al. 2010). In this context, epigenetic biomarkers could help identify which patients are more likely to benefit from specific therapies, optimizing treatment outcomes while reducing unnecessary exposure to ineffective or toxic drugs.

Emerging evidence suggests that both exercise and dietary polyphenols serve as powerful environmental modulators of the epigenome, with the potential to counteract pathological processes (Abraham et al. 2023). These lifestyle factors can reshape epigenetic imprinting established during early development, thereby reactivating protective gene expression patterns and promoting cellular resilience. By influencing key epigenetic mechanisms, including DNA methylation, histone modification, and noncoding RNA regulation, exercise and polyphenols may work synergistically to restore healthy gene regulation disrupted in neurodegenerative conditions. As such, the strategic combination of physical activity and polyphenol‐rich diets holds promise not only for modifying disease risk and progression but also for re‐establishing a more youthful and functional cellular phenotype. Targeting the epigenome through these noninvasive, modifiable interventions may represent a novel and accessible approach to preventing or slowing the onset of PD and other age‐associated neurological disorders. Given the novelty and multidisciplinary nature of research on epigenetic modulation by exercise and polyphenols in PD, we adopted a narrative review format to allow for greater flexibility in integrating mechanistic, preclinical, and early‐stage clinical data. While this approach does not follow a formal systematic review protocol, it enables us to synthesize complex and emerging insights from disparate sources. Nonetheless, we acknowledge the inherent limitations, including potential selection bias and the absence of a formal quality appraisal.

## 2 Epigenetics in PD

Epigenetic regulation comprises a complex set of molecular mechanisms that control gene expression without altering the underlying DNA sequence. Among the most studied of these is DNA methylation, which involves the transfer of a methyl group to the fifth carbon of cytosine, forming 5‐methylcytosine (Yamagata et al. 2009). This process is mediated by DNA methyltransferases (DNMTs), where DNMT3a and DNMT3b are responsible for de novo methylation, while DNMT1 maintains existing methylation patterns during cell division (Yamagata et al. 2009; Moore et al. 2013). Demethylation can occur actively, through the action of TET enzymes, or passively via replication‐dependent dilution (Moore et al. 2013; Wu and Zhang 2017). The TET enzymes catalyze a series of oxidative reactions converting 5mC into intermediates such as 5‐hydroxymethylcytosine, which is often associated with transcriptional activation (Wu and Zhang 2017). In gene promoters, hypermethylation is generally linked to gene silencing. This occurs either by preventing transcription factors from binding or by attracting methyl‐binding proteins, which then recruit chromatin remodeling complexes to repress gene activity (Jones 2012; Tate and Bird 1993; Schübeler 2015). Interestingly, methylation plays different roles depending on its genomic location. While promoter CpG sites of active genes are typically unmethylated, CpGs in gene bodies may be methylated in a tissue‐specific way (Jones 2012; Huang et al. 2015). Enhancer regions, particularly super‐enhancers, also show dynamic methylation patterns crucial for cell identity and function (Huang et al. 2011; Luo and Lin 2016). Aberrant methylation in these regions has been implicated in various diseases, including cancer (Bell et al. 2016).

**FIGURE 1:**
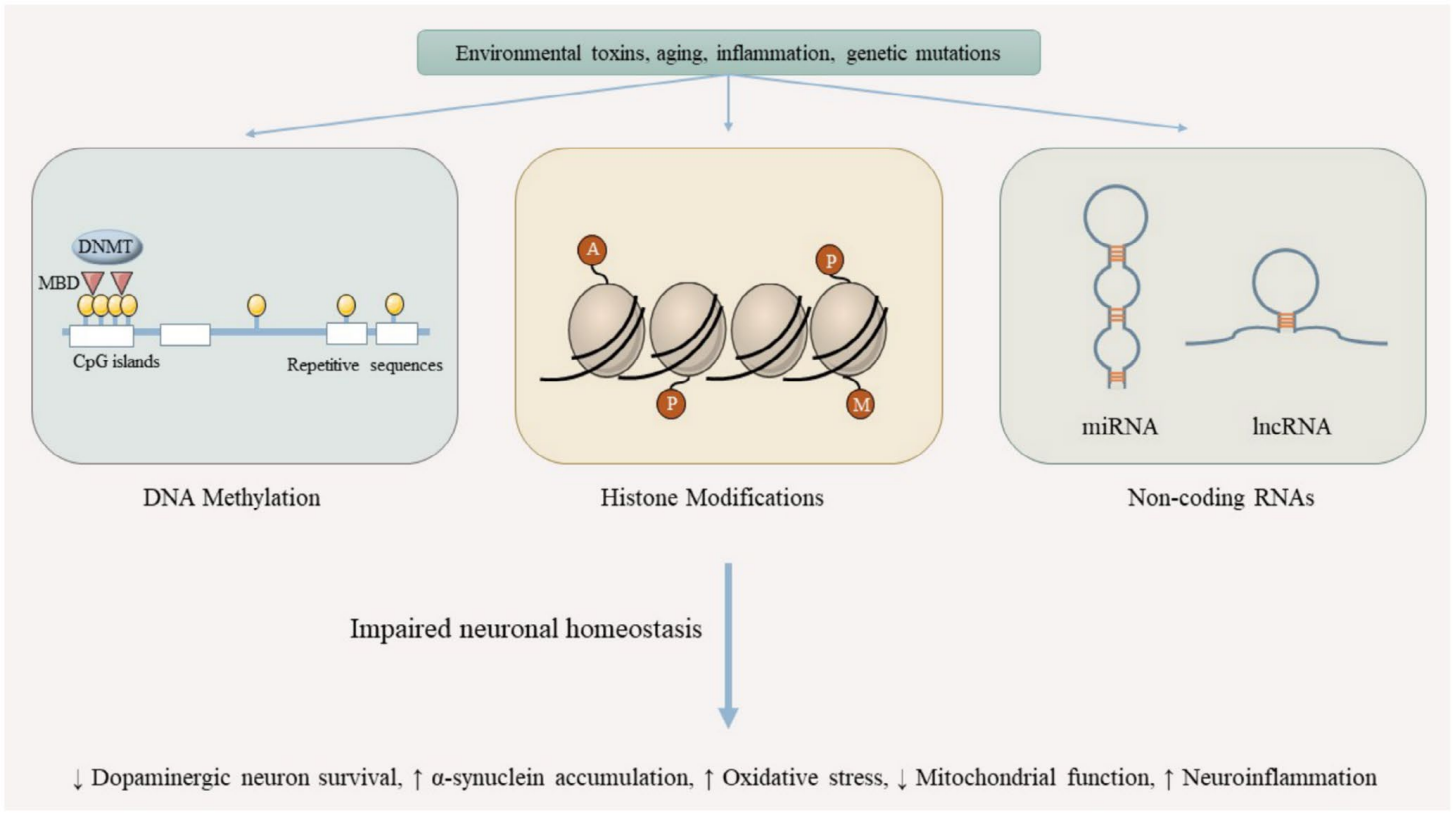
Synergistic epigenetic modulation by exercise and polyphenol intake in Parkinson's disease. This schematic illustrates how physical exercise and dietary polyphenols can converge to influence key epigenetic processes. Both interventions increase histone acetylation (notably H3 and H4), modulate microRNA expression (e.g., miR‐126, miR‐30, miR‐133b), and downregulate pro‐inflammatory miRNAs (e.g., miR‐21, miR‐146a). These epigenetic shifts contribute to neuroprotective outcomes including elevated BDNF levels, reduced inflammatory cytokines (IL‐6, TNF‐α), and improved oxidative stress profiles, potentially slowing PD progression.

Another major player in epigenetic regulation is histone posttranslational modifications (PTMs). DNA is packaged in chromatin, which is composed of nucleosomes—units of DNA wrapped around histone proteins H2A, H2B, H3, and H4 (Villeneuve and Natarajan 2010). The amino‐terminal tails of these histones can undergo PTMs such as acetylation, methylation, phosphorylation, and others, collectively forming what is referred to as the “histone code” (Javierre et al. 2010; Adcock et al. 2005). These modifications can influence chromatin structure and gene activity either by altering electrostatic interactions between histones and DNA or by recruiting reader proteins and chromatin remodelers (Urdinguio et al. 2009; Feng and Fan 2009). For example, histone acetylation—mediated by histone acetyltransferases (HATs) and removed by histone deacetylases (HDACs)—reduces the positive charge on histones, loosening chromatin and facilitating transcription factor access (Jones and Baylin 2007; Sharma et al. 2010). Active gene promoters are often marked by H3K4me3 and acetylation of histones H3 and H4, whereas gene bodies tend to feature H3K36me3 and H3K79me3 (Widschwendter et al. 2007). Conversely, repressive marks such as H3K27me3 and H3K9me3 are linked to gene silencing and are deposited by complexes such as PRC2 (Schlesinger et al. 2007). The third key mechanism is the action of noncoding RNAs (ncRNAs). These include small ncRNAs such as microRNAs (miRNAs) and piRNAs, and long noncoding RNAs (lncRNAs), both of which regulate gene expression through diverse mechanisms (Ohm et al. 2007). miRNAs bind mainly to the 3′ untranslated regions of mRNAs, blocking translation through the RNA‐induced silencing complex (Shen, Toyota, et al. 2007). lncRNAs can act as scaffolds, guides, or decoys for transcriptional machinery, influencing both DNA and RNA‐level regulation (Seligson et al. 2005). Enhancer RNAs (eRNAs), a subtype of lncRNAs, and circular RNAs (circRNAs) also modulate transcription through interactions with transcription factors and RNA polymerase II (Figueroa et al. 2010; Ellinger et al. 2010; Bachmann et al. 2006). Together, these epigenetic mechanisms form an intricate regulatory system that shapes gene expression profiles critical for both normal development and disease states, including neurodegenerative disorders such as PD (Figure 1).

## 3 Exercise and Epigenetic Reprogramming in PD

### 3.1 Exercise‐Induced Epigenetic Modifications

DNA methylation, a core epigenetic process, plays a pivotal role in regulating gene activity without altering the genetic code itself. Recent research has begun to reveal how physical activity, particularly aerobic exercise, might influence methylation patterns across the genome, potentially impacting neurodegenerative conditions such as mild cognitive impairment (MCI) and PD. In a 6‐month intervention study focusing on elderly African American individuals with MCI, researchers explored the genome‐wide DNA methylation shifts brought about by regular aerobic exercise (Ngwa et al. 2022). However, the sample was limited to a specific demographic group, which may limit generalizability. Participants in the intervention group engaged in supervised 40‐min aerobic sessions three times per week, whereas the control group performed stretching exercises. At both the beginning and end of the study, assessments were made using VO_2_ max tests and high‐throughput methylation profiling via the Infinium HumanMethylation450 BeadChip array. The aerobic group showed not only improved cardiovascular fitness but also significant alterations in DNA methylation, both increases and decreases, across numerous CpG sites. Notably, the VPS52 gene, which plays a role in intracellular protein transport and amyloid precursor protein processing, exhibited marked hypomethylation. This change corresponded with a near twofold rise in gene expression, confirmed by qRT‐PCR. Other genes showing similar exercise‐linked demethylation included SCARB1, ARTN, NR1H2, and PPP2R5D, all of which are implicated in neural function, lipid transport, and cellular signaling. These results suggest that physical activity may influence epigenetic landscapes in ways that promote neuroprotection and possibly delay cognitive decline (Ngwa et al. 2022). In a separate large‐scale study using data from the Taiwan Biobank, researchers examined the link between exercise, sex, and DNA methylation at a specific CpG site (cg17274742) in the GPNMB gene, which is associated with immune regulation and has been flagged as a potential biomarker for PD risk (Chen et al. 2023). Analyzing data from over 1400 participants and adjusting for lifestyle and metabolic factors, they found that men who exercised regularly had significantly lower methylation levels at this site compared to their non‐exercising counterparts and to women. This hypomethylation was exclusive to male participants, indicating a potential gender‐specific epigenetic response to exercise. The interaction between exercise and gender was statistically significant, reinforcing the idea that lifestyle factors may affect the epigenome in nuanced and individualized ways (Chen et al. 2023). Although the sample size was large and well‐characterized, the observational nature of the data precludes causal inference. Together, these findings suggest that regular physical activity has the potential to reshape DNA methylation profiles, particularly in genes related to neurological function and disease risk.

Emerging evidence highlights the role of histone modifications, particularly acetylation, as a crucial mechanism through which physical exercise promotes neuroprotection. Histone acetylation is primarily controlled by the dynamic balance between HATs, which promote gene transcription, and HDACs, which suppress it (Chen et al. 2015). Exercise appears to influence this balance, potentially enhancing cognitive function and neuroplasticity (Kukla‐Bartoszek and Głombik 2024). In a study examining the frontal cortex of Wistar rats, different exercise regimens were used to explore how physical activity alters the brain's epigenetic landscape. A single running session significantly increased HAT activity, while a two‐week regimen of moderate daily treadmill exercise reduced HDAC activity. Both protocols seemed to induce a state of histone hyperacetylation, suggesting enhanced transcriptional readiness in the frontal cortex, a brain region essential for higher cognitive processes. These findings underscore the brain's sensitivity to epigenetic modulation through physical activity and suggest a dual pathway through which acute and chronic exercise influence acetylation dynamics (Spindler et al. 2014).

Similarly, another study focused on the hippocampus, a region deeply involved in memory and learning. Male Wistar rats were subjected to either a one‐time treadmill session or a consistent two‐week protocol. In both conditions, histone acetylation was modulated: Single exercise sessions led to decreased HDAC activity and increased HAT activity immediately and one hour post exercise, resulting in a net increase in the HAT/HDAC ratio, an indicator of a more transcriptionally active chromatin state. Interestingly, this acetylation balance also followed circadian rhythms, with significantly lower HAT/HDAC ratios observed in the early morning compared to the afternoon. This suggests that the timing of exercise could further influence its epigenetic impact. Altogether, these data support the notion that the neuroprotective effects of exercise may be closely tied to histone acetylation, modulated by both activity and biological rhythms (Elsner et al. 2011). Maejima and colleagues took a different angle by combining aerobic exercise with pharmacological intervention. In their study, ICR mice were divided into groups receiving either treadmill training, an HDAC inhibitor (sodium butyrate), or both (Maejima et al. 2021). The administration of sodium butyrate inhibited HDAC activity and increased acetylation of histone H4, which corresponded with elevated expression of immediate early genes like *c‐fos* and *Arc*, as well as neurotrophins such as brain‐derived neurotrophic factor (BDNF) and NT‐4, genes crucial for neuroplasticity. Although exercise alone also enhanced acetylation levels of histones H3 and H4, it did not produce significant changes in gene expression. Moreover, no synergistic effects were observed when combining both interventions, suggesting that pharmacological HDAC inhibition might offer a more consistent method of promoting a neuroplastic environment than exercise alone, unless the exercise protocol is carefully optimized (Maejima et al. 2021). Together, these studies suggest that exercise‐induced changes in histone acetylation, through modulation of HAT and HDAC activity, play a vital role in enhancing brain plasticity and cognitive resilience. However, the type, duration, and timing of exercise are key factors that may shape its epigenetic effects.

Beyond its behavioral benefits, growing evidence highlights the molecular adaptations induced by exercise, particularly the regulation of ncRNAs, including miRNAs, lncRNAs, and circRNAs. These ncRNAs are key modulators of gene expression involved in synaptic function, neuronal survival, inflammation, and mitochondrial dynamics. A growing body of preclinical and clinical studies suggests that exercise modulates ncRNA profiles in ways that support neuroprotection and functional recovery in PD (Table 1). In a recent transcriptome‐wide investigation using a PD mouse model, four weeks of aerobic exercise training (AET) not only improved behavioral outcomes but also induced marked histological improvements in the substantia nigra. Large multipolar cells with prominent processes, alongside surrounding neutrophils, were observed in the AET group, indicating cellular restoration. RNA‐seq data revealed 62 differentially expressed long noncoding RNAs (DE‐lncRNAs), with 55 upregulated and 7 downregulated. Among them, LOC102633466, LOC102637865, and LOC102638670 were significantly elevated following AET. These lncRNAs were found to influence key pathways including the extracellular matrix (ECM)‐receptor interaction, Wnt signaling, and the PI3K/AKT/mTOR axis—pathways known for their roles in neuronal growth, survival, and synaptic plasticity. Quantitative PCR validated the upregulation of these lncRNAs, and a competing endogenous RNA (ceRNA) network analysis suggested that these molecules may act as miRNA sponges, thereby indirectly regulating gene expression critical for neuronal repair (Zhang et al. 2020).

**TABLE 1:** Studies investigating the role of exercise training in affecting noncoding RNAs in PD.

ncRNAs investigated	Exercise protocol	Model	Key findings	Associated pathways/targets	Refs.
miR‐3557 (↑), miR‐324 (↓)	8 weeks regular aerobic exercise before 6‐OHDA‐induced lesion	Rat PD model	Exercise reduced apomorphine‐induced rotations, preserved striatal structure, increased TH, reduced α‐synuclein; miR‐3557/324 expression modulated	CaMK2α (↑), CaMKV (↓), Vdac1 (↓), PI3K/mTOR (↑), UCH‐L1 (↑)	Rashidfard et al. (2024)
miR‐3557 (↑), miR‐324 (↓)	8 weeks of regular aerobic exercise (swimming)	6‐OHDA‐induced PD model in rats	Exercise altered expression of miR‐3557 and miR‐324, improved behavior (reduced rotations), increased TH, reduced α‐synuclein, and enhanced neuroprotection	CaMKII↑, CaMKV↓, Vdac1↓, PI3K/mTOR↑, UCH‐L1↑	Liu et al. (2019)
11 miRNAs (e.g., miR‐320c, miR‐181a‐2‐3p, miR‐619‐5p)	3‐month home‐ and community‐based exercise program (aerobic + dance, 5×/week)	Human PD patients (*n* = 19)	Exercise led to motor improvements (UPDRS, 6MWT, TUG); 10 miRNAs upregulated, 1 downregulated post‐intervention	MAPK, Wnt, and Hippo signaling pathways; GO terms: binding, catalytic activity, transcription regulation	Zhang et al. (2024)
miR‐106a‐5p, miR‐103a‐3p, miR‐29a‐3p	Interval training on cycle ergometer (30 min, 3×/week, 8 weeks)	Human, male PD patients (*n* = 8)	Significant upregulation of miR‐106a‐5p after training; miR‐103a‐3p and miR‐29a‐3p showed positive correlations with MMSE scores; cognitive improvement associated with miRNA expression changes	Wnt signaling pathway, PI3K/AKT (via PIK3R1), MEF2D, GPR37, DKK1, HIF‐1α, ATG7	Da Silva et al. (2021)
LOC102633466, LOC102637865, LOC102638670, LOC102633419, LOC102634932, LOC102637640	Aerobic exercise training (grasping, rotation, walking, and balance training for 4 weeks; 30 min/day, 6 days/week)	MPTP‐induced Parkinson's disease mouse model (C57BL/6J male mice, 10–12 weeks old)	Exercise improved motor function and pathological features of PD. 62 differentially expressed lncRNAs were identified; 55 upregulated and 7 downregulated in AET group versus PD	lncRNA–miRNA–mRNA ceRNA network; ECM‐receptor interaction, Wnt signaling, PI3K/AKT/mTOR pathway; targets include Col6a1 and Wnt6	Zhang et al. (2020)

In another quasi‐experimental study involving male PD patients, the impact of an 8‐week interval training regimen on circulating miRNA levels and cognitive function has been examined. Participants in the exercise group demonstrated a significant upregulation of miR‐106a‐5p, miR‐103a‐3p, and miR‐29a‐3p compared to controls. These miRNAs have been previously associated with neurogenesis, anti‐inflammatory activity, and synaptic maintenance. The observed elevation in miRNA levels was positively correlated with cognitive improvements post‐intervention, indicating a molecular underpinning for the neurocognitive benefits of physical activity in PD. Despite limitations such as small sample size and lack of randomization, this study offers compelling evidence for the regulatory role of miRNAs in exercise‐induced cognitive enhancement (Da Silva et al. 2021). Additional clinical insights were obtained from a study employing a three‐month home‐ and community‐based exercise program in 13 PD patients, with six serving as non‐exercising controls. Notable improvements were recorded across various functional metrics, including the Unified Parkinson's Disease Rating Scale (UPDRS), Six‐Minute Walk Test, Timed Up and Go, and the Berg Balance Scale. Small RNA sequencing revealed 11 significantly altered miRNAs post‐intervention, with 10 being upregulated. Functional enrichment analyses linked the targets of these miRNAs to pivotal pathways such as Wnt, Hippo, and MAPK signaling, along with processes involving catalytic activity and transcriptional regulation. These results align with the hypothesis that exercise reshapes the miRNA transcriptome to support neuroplasticity and functional recovery in PD (Zhang et al. 2024).

In rodent models, additional insights into the molecular mechanisms were gained through the analysis of miRNA signaling in response to aerobic training. In a study involving rats subjected to 6‐hydroxydopamine (6‐OHDA)‐induced lesions, eight weeks of treadmill running led to reduced rotational behavior and preservation of neuronal ultrastructure. Notably, miR‐3557 expression was upregulated while miR‐324 was downregulated in the exercised animals. This miRNA profile was associated with increased levels of CaMK2α and decreased expression of CaMKV and Vdac1, components of the CaMK signaling pathway. Furthermore, exercise enhanced the expression of PI3K, mTOR, and UCH‐L1, proteins associated with cell survival and protein degradation, suggesting that exercise‐induced miRNAs help to coordinate signaling networks that enhance neuronal resilience and mitigate PD pathology (Liu et al. 2019). In another experiment using a reserpine‐induced PD rat model, six weeks of high‐intensity interval swim training produced molecular changes in hippocampal tissue. The expression of mir‐874, significantly elevated in PD rats (*p* = 0.001 vs. control), was normalized following exercise (*p* = 0.02 vs. trained), while DJ‐1, a neuroprotective gene downregulated in PD (*p* = 0.02 vs. control), was restored in the exercise group (*p* = 0.04 vs. trained). These findings indicate that physical training may reverse disease‐associated redox imbalances and neuronal stress responses through miRNA regulation. Additional research in a similar model showed that exercise reduced hippocampal levels of let‐7 and miR‐23b, miRNAs implicated in neuroinflammation and synaptic regulation, further supporting the anti‐inflammatory and neurorestorative capacity of exercise in PD (Rashidfard et al. 2024).

Fan et al. (2022) have also reported that in an MPTP‐induced PD mouse model, four weeks of treadmill running led to the differential expression of 142 circRNAs between sedentary PD mice and those subjected to exercise. Specifically, circZfp827 and circTshz2 were elevated in the PD state but downregulated following exercise, whereas circHivep2 showed the opposite pattern. Bioinformatic analyses implicated these circRNAs in regulating dopaminergic synapse function and calcium signaling. Experimental validation using dual‐luciferase assays confirmed that circTshz2 directly sponged mmu‐miR‐326‐3p to regulate tyrosine hydroxylase, a key enzyme in dopamine synthesis. These data reveal that exercise may restore dopaminergic function through complex circRNA–miRNA–mRNA regulatory circuits (Fan et al. 2022). Taken together, these findings underscore the multifaceted impact of physical exercise on the noncoding RNA landscape in PD. From the regulation of lncRNA‐mediated ceRNA networks to the miRNA‐driven modulation of survival pathways and circRNA control of dopaminergic gene expression, exercise emerges as a potent molecular modulator. Consequently, ncRNAs represent not only potential biomarkers for monitoring the efficacy of exercise interventions but also attractive targets for therapeutic modulation aimed at halting or reversing PD progression.

It is important to note, however, that not all studies have reported significant epigenetic alterations in response to exercise. For instance, in a study on elderly women, although improvements in cognitive performance and inflammatory markers were observed, histone acetylation changes did not reach statistical significance, suggesting that individual variability, duration, and intensity of training may all influence epigenetic responsiveness (Henrique et al. 2023). Moreover, some DNA methylation changes detected after aerobic training in cognitively impaired adults were subtle and gene‐specific, and did not always translate into functional gene expression shifts (Ngwa et al. 2021). These inconsistencies highlight the complexity of capturing epigenetic dynamics and the need for standardized protocols and biomarkers.

### 3.2 Neuroprotective Epigenetic Targets of Exercise

In addition to broad epigenetic alterations, specific molecular targets have emerged as key mediators of the neuroprotective effects of exercise in PD. These include genes and transcription factors involved in neuronal survival, mitochondrial function, and oxidative stress response. This section highlights three such targets, BDNF, peroxisome proliferator‐activated receptor gamma coactivator‐1 alpha (PGC‐1α), and nuclear factor erythroid 2‐related factor 2 (Nrf2), and examines how their expression is influenced by exercise through epigenetic mechanisms such as histone acetylation and DNA methylation (Table 2).

**TABLE 2:** Neuroprotective targets of exercise in PD via epigenetic modulation.

Target	Function	Epigenetic mechanism	Exercise effect	Evidence source	Refs.
BDNF	Neurotrophic support	Histone acetylation ↑	↑ BDNF mRNA/protein	Rodent & human studies	Fraga et al. (2021)
PGC‐1α	Mitochondrial biogenesis	Histone remodeling	↑ Expression	Transcriptomics/meta‐analysis	Santos et al. (2014)
Nrf2	Antioxidant defense	Promoter demethylation	↑ TFAM, NQO1	Rotenone model + DNA methylation data	Chen, Zhu, et al. (2021)

#### 3.2.1 BDNF

Exercise is a potent modulator of neuroplasticity and cognitive function, and BDNF plays a pivotal role in mediating these effects. In the context of PD, where neurodegeneration and cognitive decline are prominent, strategies that enhance BDNF expression may offer therapeutic benefit. Evidence suggests that the effects of exercise on BDNF are at least partly mediated by epigenetic mechanisms, particularly through histone acetylation.

Histone acetylation alters chromatin structure, enabling transcription factors easier access to gene promoters such as those of BDNF. This modification is dynamically controlled by HATs and HDACs. A series of rodent and human studies highlight how exercise influences this regulatory axis to promote BDNF transcription. In senescence‐accelerated mice (SAMP1), four weeks of treadmill running (60 min/day, 5 days/week) led to significantly improved cognitive performance and elevated hippocampal BDNF mRNA levels. Interestingly, exercise upregulated both HAT and HDAC activities, indicating a remodeling of chromatin structure to support enhanced gene transcription. Notably, expression of the p75 neurotrophin receptor, known for its pro‐apoptotic function, was downregulated, suggesting that exercise shifts the neurotrophic balance toward neuroprotection and synaptic plasticity (Maejima et al. 2018).

Pharmacological inhibition of HDACs has been shown to amplify the cognitive and molecular benefits of exercise. In a study where mice were administered sodium butyrate (NaB), an HDAC inhibitor, and concurrently engaged in treadmill running (10 m/min, 60 min/day for 4 weeks), combined treatment produced superior improvements in learning and memory compared to either intervention alone. NaB treatment significantly increased BDNF and receptor gene expression in the hippocampus, and this was further potentiated by exercise. These findings underscore the importance of HDAC suppression in facilitating exercise‐induced BDNF upregulation and suggest a possible therapeutic synergy in combining epigenetic modulators with physical activity in PD (Kitahara et al. 2021). Beyond pharmacological agents, exercise itself can induce endogenous HDAC inhibition through metabolic intermediates. β‐hydroxybutyrate (β‐HB), a ketone body generated during prolonged aerobic exercise, was found to activate BDNF promoter I transcription in the mouse hippocampus. Mechanistically, β‐HB suppresses HDAC2 and HDAC3 activity, enhancing histone acetylation at BDNF gene loci. This leads to elevated BDNF expression and increased synaptic activity via TrkB signaling. This endogenous epigenetic pathway offers a compelling mechanistic explanation for how prolonged exercise exerts neurotrophic effects (Sleiman et al. 2016).

Human studies support the relevance of these findings in aging populations. In a Brazilian cohort of institutionalized older adults, an 8‐week multimodal exercise regimen (2 sessions/week, 60 min each) improved cognitive scores and mobility. While BDNF levels did not significantly change, there was a notable increase in histone H3 acetylation in peripheral blood cells, suggesting that chromatin remodeling may underlie the functional improvements observed (Fraga et al. 2021). Similarly, a 6‐week intervention involving either conventional exercise or exergaming in elderly women resulted in cognitive enhancement and increased serum BDNF levels. Histone acetylation (H3 and H4) also rose significantly, alongside pro‐inflammatory cytokines like IL‐6 and TNF‐α. These findings reinforce the concept that histone acetylation is a critical epigenetic mediator of exercise‐induced neurotrophic and cognitive benefits, even in aging individuals who are at elevated risk for neurodegenerative conditions such as PD (Henrique et al. 2023). A recent meta‐analysis consolidates the impact of exercise on BDNF levels specifically in PD. Analyzing five randomized controlled trials with 216 patients, the study found a large pooled effect size for exercise on serum BDNF. Moreover, improvements were noted in core clinical metrics including the UPDRS, Berg Balance Scale, and 6‐Minute Walk Test. Although these studies did not directly assess histone acetylation, the robust elevation in BDNF levels in response to exercise strongly suggests an underlying epigenetic component (Kaagman et al. 2024).

#### 3.2.2 PGC‐1α

One of the key regulators of mitochondrial function and cellular energy metabolism, PGC‐1α, has emerged as a significant molecular target in PD. A comprehensive meta‐analysis integrating transcriptomic data from over 400 PD patients and controls identified marked downregulation of genes involved in mitochondrial bioenergetics, many of which are directly regulated by PGC‐1α. This suppression was evident even in the early stages of the disease, highlighting impaired mitochondrial electron transport, glucose metabolism, and energy sensing as early pathophysiological events in PD. Importantly, experimental models demonstrated that stimulating PGC‐1α expression counteracted dopaminergic neuron loss triggered by toxic stimuli such as mutant α‐synuclein and the pesticide rotenone, underscoring its neuroprotective potential. These findings suggest that restoring PGC‐1α activity may serve as a promising strategy to prevent or slow neurodegeneration in PD (Zheng et al. 2010). Interestingly, PGC‐1α is also a critical factor in skeletal muscle metabolism and is epigenetically regulated through histone modifications. In insulin‐resistant states such as type 2 diabetes, PGC‐1α expression is diminished, accompanied by altered histone marks that suppress mitochondrial gene programs. However, regular aerobic exercise has been shown to reverse this pattern by increasing PGC‐1α levels, thereby enhancing mitochondrial density and the expression of glucose transporters such as GLUT4. Though these observations are rooted in metabolic disease research, they underscore a shared epigenetic mechanism where exercise‐induced PGC‐1α upregulation restores cellular energy balance (Santos et al. 2014). By extension, it is plausible that similar exercise‐induced epigenetic modulation of PGC‐1α in the brain contributes to its neuroprotective effects in PD. The ability of exercise to activate PGC‐1α may not only restore mitochondrial function but also offset neurotoxic insults associated with PD progression. These insights position PGC‐1α as a key mediator linking lifestyle interventions, particularly physical activity, to epigenetically regulated neuroprotection in PD.

#### 3.2.3 Nrf2

Nrf2 has emerged as a pivotal transcription factor in the body's defense against oxidative stress, a key player in PD pathology. Exercise appears to engage this pathway, offering neuroprotection by enhancing the expression of genes under the control of the antioxidant response element (ARE). Studies in PD animal models have shown that treadmill‐based aerobic training not only improves behavioral deficits such as bradykinesia, impaired gait, and motor coordination, but also triggers upregulation of genes downstream of the Nrf2 pathway, namely TFAM, Nrf2, and NQO1. Interestingly, exercise alone provided more comprehensive cognitive and motor benefits than L‐dopa, and their combination yielded even broader symptom relief, suggesting additive or synergistic effects through distinct mechanisms (Monir et al. 2020). Mechanistically, regular exercise enhanced mitochondrial resilience and biogenesis within the striatum, particularly against neurotoxins like 6‐OHDA and nitric oxide donors. These findings reinforce the idea that the Nrf2–ARE signaling axis is actively involved in mediating exercise‐induced neuroprotection in dopaminergic pathways. Furthermore, activation of this pathway has been associated with resistance to oxidative damage, one of the central factors driving PD progression (Aguiar et al. 2016).

From an epigenetic standpoint, there is growing recognition that Nrf2 transcription is regulated by DNA methylation, particularly in promoter regions (Zhao et al. 2018). While this has been more thoroughly described in cancer and metabolic diseases, emerging evidence suggests a similar mechanism may operate in neurodegeneration (Guo et al. 2015). Exercise has been shown to reverse promoter hypermethylation of Nrf2 in models of osteoporosis, thereby restoring antioxidant defense gene expression (Chen, Zhu, et al. 2021). Although direct evidence of exercise reversing Nrf2 methylation in PD remains limited, these findings raise the possibility that physical activity modulates Nrf2 expression, at least in part, through epigenetic remodeling. Taken together, the body of evidence suggests that Nrf2 activation by exercise may serve as a crucial epigenetically regulated mechanism underlying its neuroprotective benefits in PD. Further investigation is warranted to clarify whether exercise can directly modify Nrf2 methylation patterns in PD‐affected brain regions.

## 4 Epigenetic Effects of Polyphenols in PD

Polyphenols represent a structurally diverse class of secondary plant metabolites, broadly categorized into flavonoids, stilbenes, phenolic acids, and lignans. This chemical diversity profoundly influences their biological activity, including their interaction with the epigenome. For instance, flavonoids such as epigallocatechin gallate (EGCG) tend to inhibit DNMTs and histone deacetylases, whereas stilbenes like resveratrol activate sirtuins and modulate chromatin accessibility. These distinct pathways reflect differences in molecular structure, absorption, metabolism, and cellular uptake. Consequently, the epigenetic effects observed across studies are often heterogeneous, making direct comparisons difficult and generalizations about “polyphenols” potentially misleading. This variability underscores the importance of evaluating each compound on its own mechanistic terms, particularly in the context of complex diseases such as PD. Therefore, this section focuses on three well‐characterized polyphenols, resveratrol, curcumin, and EGCG, and summarizes their documented epigenetic actions in PD or closely related neurodegenerative models.

### 4.1 Resveratrol

Resveratrol, a stilbene found in red grapes and peanuts, has demonstrated neuroprotective potential in PD models through both direct and indirect epigenetic regulation. One of its most studied mechanisms is the activation of SIRT1, a class III histone deacetylase involved in neuronal survival and mitochondrial function. SIRT1 activation by resveratrol promotes deacetylation of histones at promoter regions of neurotrophic genes like BDNF and FOXO3a, enhancing their transcription and supporting neuronal resilience (Lagouge et al. 2006). In a mouse model of PD induced by MPTP, resveratrol administration improved locomotor behavior and preserved dopaminergic neurons in the substantia nigra, effects partially mediated through SIRT1‐dependent deacetylation of p53 and histone H3 (Jin et al. 2008). Resveratrol has also been shown to alter non‐coding RNA expression. For instance, it modulates miR‐132 and miR‐124 levels, both of which are implicated in neuroinflammation and synaptic maintenance (Zhang et al. 2019; Li et al. 2025). While human studies are still lacking, these findings highlight resveratrol's dual capacity to affect transcriptional programs via histone modifications and to fine‐tune posttranscriptional regulation via miRNAs.

### 4.2 Curcumin

Curcumin, the principal curcuminoid in turmeric, has long been studied for its anti‐inflammatory and antioxidant effects. More recently, it has emerged as a potent epigenetic modulator. In the context of PD, curcumin's influence on DNA methylation and miRNA regulation has gained attention. Studies found that curcumin supplementation reversed aberrant expression of miR‐30a and miR‐874, two miRNAs involved in autophagy and neuronal apoptosis. These changes were associated with reduced expression of caspase‐3 and normalization of redox‐sensitive pathways, suggesting a restoration of epigenetic homeostasis (Bhattacharjee and Dashwood 2020; Khor et al. 2011; Rathore et al. 2023). Although these experiments were conducted in arsenic‐exposed rodent models, the affected pathways, namely oxidative stress, apoptosis, and redox‐sensitive miRNA regulation, are also central to PD pathogenesis. Still, caution is advised in directly translating these findings to PD due to differences in disease etiology and cellular targets.

Curcumin has also been shown to inhibit DNMTs, leading to hypomethylation of promoters for genes involved in neuroprotection, including Nrf2 and HO‐1 (Khor et al. 2011). In a PD‐like rat model, curcumin elevated Nrf2 levels and improved motor function, likely through demethylation of the Nrf2 promoter and activation of its downstream antioxidant targets (Cui et al. 2016). These effects are particularly relevant given the growing interest in oxidative stress regulation as a target in PD pathogenesis. Furthermore, curcumin can influence histone acetylation, although studies specific to PD remain limited. Its ability to alter chromatin accessibility may underlie the observed upregulation of synaptic genes in neurodegenerative contexts.

### 4.3 EGCG

EGCG, the most abundant catechin in green tea, is another polyphenol with known epigenetic activity. In cancer and metabolic models, EGCG inhibits DNMT1 and class I HDACs, thereby promoting a more transcriptionally active chromatin state (Li et al. 2022). In neurodegenerative research, EGCG has shown potential in protecting against dopaminergic neurodegeneration by modulating epigenetic regulators. In a study, EGCG restored mitochondrial membrane potential and decreased reactive oxygen species while simultaneously upregulating miR‐200b, a microRNA involved in oxidative stress response (Fang et al. 2003). This in vitro model captures specific oxidative stress pathways but lacks the complexity of in vivo neural environments.

Additionally, EGCG exposure resulted in decreased expression of DNMT1 and HDAC2, leading to global DNA hypomethylation and increased acetylation at the promoters of antioxidant genes such as SOD2 and GPX1 (Martínez‐Iglesias et al. 2025). Although these findings have not yet been confirmed in human PD trials, they reinforce EGCG's role as a pleiotropic epigenetic modulator with relevance to neurodegenerative disease pathways. While most epigenetic studies on EGCG originate from cancer or metabolic models, the conserved nature of its interactions with DNMTs and HDACs justifies exploration in PD. Nonetheless, tissue‐specific epigenomic responses and bioavailability limitations may alter its effects in neuronal contexts.

Taken together, these findings provide a compelling rationale for targeting the epigenome with dietary polyphenols in PD. Each compound, resveratrol, curcumin, and EGCG, acts through multiple molecular pathways, but converges on a few common epigenetic mechanisms: promotion of histone acetylation, inhibition of DNA methylation, and modulation of disease‐relevant miRNAs. These actions support the expression of neurotrophic factors (e.g., BDNF), antioxidant enzymes (e.g., Nrf2, SOD2), and anti‐apoptotic pathways (e.g., FOXO3a), all of which are dysregulated in PD. While resveratrol, curcumin, and EGCG have demonstrated epigenetic activity in preclinical models, the translation of these findings into consistent human outcomes has proven difficult. Several clinical studies, for instance, have failed to detect meaningful changes in histone acetylation or DNA methylation following polyphenol supplementation, likely due to issues of bioavailability, metabolic breakdown, and low target tissue penetration (Fang et al. 2003; Dani et al. 2021). These limitations underscore the importance of delivery strategies and model selection in polyphenol research.

## 5 Combined Effects of Exercise and Polyphenols on Epigenetics

Physical exercise and dietary polyphenols are recognized as potent modulators of epigenetic mechanisms. Exercise can alter patterns of DNA methylation, modify histones, and regulate ncRNAs in ways that support brain health. Similarly, polyphenols (e.g., resveratrol and other flavonoids) influence epigenetic marks such as histone acetylation and DNA methylation, and can affect microRNA expression. These changes often adjust gene expression in pathways related to neuronal function, stress responses, and survival. For example, both exercise and polyphenols can promote the expression of genes involved in neuronal resilience.

Crucially, exercise and polyphenols also share overlapping molecular effects. Both interventions reduce oxidative stress and modulate inflammatory signaling. Since oxidative damage and chronic inflammation are key drivers of PD pathology, targeting these processes could influence disease course. While the individual benefits of exercise and polyphenol‐rich diets on neural health and epigenetic regulation are well‐documented, their combined impact on the epigenetic landscape in PD remains largely unexplored. Investigating this interaction could reveal new strategies to modulate disease‐relevant gene expression in PD. The potential synergy between physical exercise and polyphenol‐rich compounds in PD is gaining research attention. One study by Askar et al. (2019) explored how ferulic acid (FR), an antioxidant polyphenol, and swimming‐based physical activity impacted PD symptoms induced by rotenone in mice. While both interventions individually improved motor behavior, enhanced tyrosine hydroxylase expression, and reduced alpha‐synuclein aggregation in the striatum, the combined treatment did not outperform FR alone. The data suggest that although exercise contributes to neuroprotection, FR may exert a more potent influence on its own, particularly through upregulating protective proteins like Hsp70. Nonetheless, this study supports the idea that both exercise and polyphenols can target PD‐related pathophysiology.

Regarding studies focused specifically on epigenetic mechanisms, one clinical trial involving individuals with PD examined the effect of a four‐week aquatic exercise program with or without daily grape juice consumption. While both groups showed functional improvements, better mobility, reduced fall risk, and enhanced endurance, the analysis also revealed increased blood levels of BDNF and histone H4 acetylation after the intervention. Interestingly, the addition of grape juice did not yield further gains, indicating that exercise alone may be sufficient to induce these beneficial epigenetic and neurotrophic changes. The upregulation of histone acetylation suggests that exercise might influence chromatin accessibility and gene expression relevant to neuronal plasticity (Oliveira et al. 2020). While the clinical setting adds translational value, the study was underpowered and lacked a placebo‐controlled design. Another study conducted on individuals with Alzheimer disease demonstrated that aerobic training combined with resveratrol and fisetin supplementation markedly boosted hippocampal expression of neurogenesis‐related genes, BDNF, VEGF, and FGF7 in mice injected with amyloid‐beta. These findings hint at shared pathways in neurodegenerative diseases, showing how polyphenols and physical activity might promote neural repair and synaptic function through gene regulation (Jahanbakhsh et al. 2024). Although this study was conducted in the context of Alzheimer's disease, the examined pathways, such as BDNF upregulation and synaptic plasticity, are also disrupted in PD. Nonetheless, given the differences in disease pathology and progression, extrapolation of these effects to PD requires careful consideration.

Another related study extended this concept to a healthy aging population, combining red grape juice intake with concurrent physical training. Although the intervention did not lead to measurable changes in global H3 and H4 acetylation, both the exercise‐only and the combined groups showed a significant decrease in IL‐6, a marker of inflammation. Additionally, improvements were noted in oxidative stress parameters and antioxidant enzyme activity. Notably, the group receiving grape juice without exercise showed a rise in non‐enzymatic antioxidant defenses, likely due to its polyphenol content. Despite the lack of epigenetic shifts, the anti‐inflammatory and oxidative stress modulations suggest an indirect route by which polyphenol‐exercise synergy could benefit brain health (Dani et al. 2021). However, as this study did not involve individuals with PD or dopaminergic dysfunction, these findings should be interpreted as preliminary, offering insight into general neuroprotective mechanisms rather than disease‐specific epigenetic effects.

In summary, while the combination of exercise and polyphenol intake appears to improve motor function, neurotrophic signaling, and markers of oxidative stress or inflammation, the current evidence does not robustly support significant epigenetic alterations, particularly in histone acetylation, when these interventions are combined. Some studies reported increases in histone H4 acetylation or BDNF levels with exercise alone, but the addition of polyphenols such as grape juice did not consistently enhance these effects. In healthy elderly subjects, even the combined protocol failed to produce changes in global histone H3 and H4 acetylation levels. Thus, while exercise and polyphenols individually influence several biological pathways linked to neuroprotection, their synergistic impact on epigenetic regulation remains limited or inconclusive, warranting further targeted investigation in the context of PD.

In PD, early dopaminergic degeneration in the substantia nigra is often well underway before clinical symptoms appear. Recent studies have pointed to miRNAs as potential biomarkers for early detection and disease progression. In a chronic MPTP‐induced PD mouse model, four miRNAs, miR‐19b, miR‐124, miR‐126a, and miR‐133b, were significantly downregulated, suggesting their relevance in PD pathophysiology and as potential therapeutic targets (Rosas‐Hernandez et al. 2018). Notably, miR‐126 plays roles in regulating neurotoxic and neuroprotective signaling, especially through PI3K/AKT and MAPK/ERK pathways, making it a promising candidate for neurodegenerative disease intervention (Briggs et al. 2015). In support of this, a study in breast cancer‐bearing mice examined the effects of endurance training and curcumin supplementation on miR‐126 expression. The combination therapy notably increased miR‐126 levels while downregulating angiopoietin‐1, with a stronger effect seen in the dual‐treatment group compared to either intervention alone. Although this study was not PD‐specific, the shared molecular pathways involved in angiogenesis and neuroprotection suggest that similar modulation could benefit PD (Kouchaki Langroudi et al. 2020). Similarly, miR‐133b, which has been implicated in PD and α‐synuclein regulation, was found to be upregulated following either soy isoflavone supplementation or high‐intensity interval training (HIIT) in an ovariectomized rat model (Nelson et al. 2018; Mirheidari et al. 2022). Interestingly, exercise alone produced the most pronounced effect, implying that physical activity may directly influence miR‐133b expression even more effectively than combined interventions in some contexts (Mirheidari et al. 2022).

**FIGURE 2:**
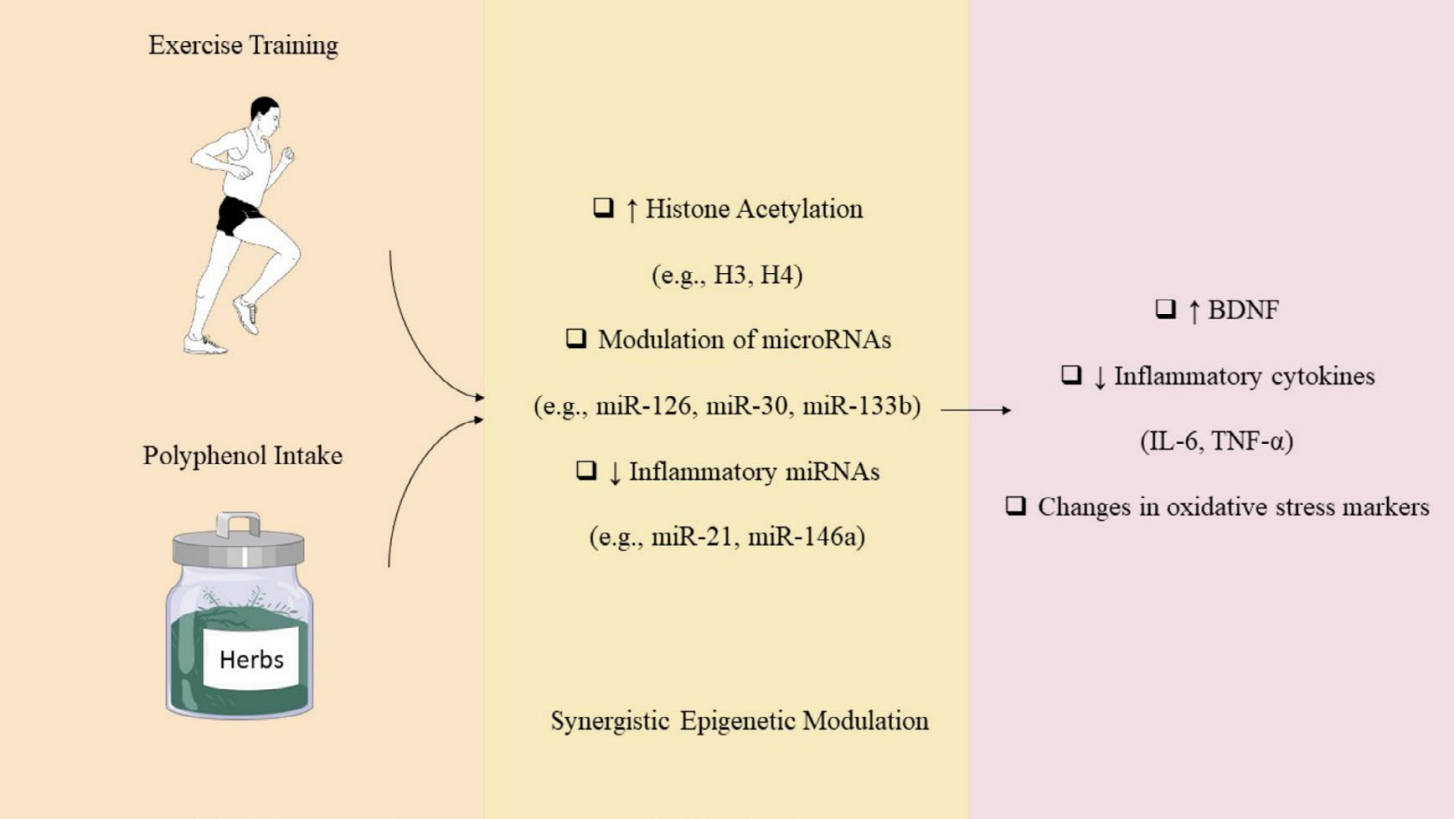
Epigenetic dysregulation in Parkinson's disease and its impact on neuronal homeostasis. This diagram summarizes the major epigenetic mechanisms implicated in PD, including DNA methylation, histone modifications (acetylation, phosphorylation, methylation), and ncRNAs (miRNAs and lncRNAs). Environmental toxins, aging, inflammation, and genetic mutations disrupt these processes, leading to impaired gene regulation. The resulting epigenetic alterations contribute to decreased dopaminergic neuron survival, increased α‐synuclein accumulation, oxidative stress, mitochondrial dysfunction, and neuroinflammation—hallmarks of PD pathology.

Another PD‐relevant miRNA, miR‐30a‐5p, was identified as a potential plasma biomarker in L‐dopa‐treated PD patients. This miRNA has been associated with mitochondrial regulation and autophagy, both critical to neuronal survival (Schwienbacher et al. 2017; Serafin et al. 2015). While not tested in a PD model, curcumin and HIIT interventions in arsenic‐exposed rats increased cardiac miR‐30 expression and improved heat shock protein levels (Mahdavi et al. 2022). Although extrapolation to PD requires caution, these results suggest curcumin may support mitochondrial integrity via miR‐30a modulation. Further supporting the role of miR‐30, a separate study on arsenic‐induced cardiac damage found that curcumin, but not HIIT, could restore miR‐30 expression and reduce apoptotic marker caspase‐3 (Majidi et al. 2022). Although mechanistically relevant, this model reflects arsenic neurotoxicity rather than PD pathology per se. These findings hint that while polyphenols like curcumin have a clear regulatory role on this miRNA, exercise alone may not suffice under certain stress conditions. Yet, this does not negate the potential synergy in other models, especially those more directly tied to neurodegeneration. Still, such findings offer a rationale for investigating how combined non‐pharmacological strategies might normalize miRNA expression patterns and influence neurodegenerative trajectories (Figure 2).

## 6 Future Directions and Clinical Implications

Despite evidence that exercise and dietary polyphenols can protect neurons, important gaps remain in understanding how these lifestyle factors modulate PD‐related epigenetics. Few studies have mapped how exercise or nutrients alter DNA methylation or histone modification in PD (Pan et al. 2013; Stephenson et al. 2021). Likewise, critical PD‐associated regulators have not been studied under combined interventions. For example, PD dopamine neurons exhibit increased miR‐126 (which impairs IGF‐1/PI3K signaling), and elevated miR‐133b (with miR‐221) distinguishes early PD (Kim et al. 2014; Chen, Deng, et al. 2021). Additionally, miR‐30a‐5p and miR‐193b‐3p have been linked to glutamate toxicity and PGC‐1α dysregulation, respectively (Meng et al. 2021; Mesarosova et al. 2024). None of these miRNA–PGC‐1α–BDNF pathways has been examined under combined exercise–polyphenol interventions. One of the main challenges research faces in this field is the translational challenges. Most evidence comes from animal and cell models. Human trials are few and underpowered. For instance, one small RCT in PD found that 4 weeks of aquatic exercise improved mobility and increased plasma BDNF and global histone H4 acetylation, but adding grape juice did not augment these effects (Oliveira et al. 2020). Clinical studies must navigate PD heterogeneity (e.g., subtypes, medications) and ensure adherence. Crucially, PD has no objective biomarker of progression, making it difficult to gauge disease‐modifying effects (Stephenson et al. 2021). On the other hand, to bridge lab and clinic, future studies should be randomized, with factorial arms (exercise + polyphenol vs. exercise + placebo vs. control) and extended follow‐up (≥ 6–12 months to capture epigenetic remodeling) (Oliveira et al. 2020; Sleiman et al. 2016). Key considerations include:

### 6.1 Control Groups

Use active controls (e.g., stretching or low‐impact exercise) and placebo beverages in the polyphenol arm.

### 6.2 Epigenetic Endpoints

Incorporate molecular biomarkers. For example, measure DNA methylation (global or PD‐gene‐specific), histone acetylation in blood cells, and circulating miRNAs (e.g., miR‐133b, miR‐30a, miR‐126) (Kim et al. 2014; Meng et al. 2021). For instance, exercise was recently linked to hypomethylation of a PD‐risk CpG (GPNMB) in men, suggesting such markers can capture lifestyle effects (Chen et al. 2023).

### 6.3 Clinical Outcomes

Combine standard PD scales (e.g., MDS‐UPDRS motor, cognition, gait metrics) with molecular readouts. The SPARX3 exercise trial, for instance, includes serum BDNF as a secondary endpoint; future trials should likewise incorporate epigenetic markers alongside clinical measures (Patterson et al. 2022).

### 6.4 Cohort Selection

Focus on early or prodromal PD (to test disease modification) and control for confounders. Consider stratification by sex or genotype, as epigenetic responses may differ (e.g., a sex‐specific effect on GPNMB methylation has been reported) (Chen et al. 2023).

Integrating epigenetic biomarkers into clinical endpoints could transform PD research. Such markers may change before symptoms and provide sensitive indicators of biological effect (Pan et al. 2013; Stephenson et al. 2021). In summary, well‐designed exercise + polyphenol trials with epigenetic readouts will be essential to determine whether lifestyle strategies can beneficially reprogram the PD epigenome and modify disease trajectory.

While direct evidence for epigenetic synergy between exercise and polyphenols in PD remains limited, there are plausible mechanistic hypotheses worth exploring. One potential avenue involves the priming of chromatin by exercise‐induced histone acetylation, which increases transcriptional accessibility at promoters of neuroprotective genes such as BDNF and Nrf2. In this more permissive epigenetic landscape, polyphenols, many of which inhibit HDACs or activate sirtuins, may further sustain or amplify gene expression by preventing chromatin recondensation. Conversely, polyphenols might first modulate microRNA expression or DNA methylation profiles, thereby setting the stage for more efficient transcriptional activation by subsequent exercise. For example, miRNAs such as miR‐30a and miR‐133b, known to be regulated by both exercise and polyphenols, could serve as convergence points in signaling cascades that enhance neuronal resilience. These speculative pathways provide a useful conceptual framework for future hypothesis‐driven research aimed at identifying true mechanistic synergy.

## 7 Conclusions

As the global burden of PD continues to rise, there is a growing need to explore non‐pharmacological strategies that go beyond symptom control and address the molecular aspects of the disease. This narrative review brings together existing evidence on how exercise and polyphenols, each well‐recognized for their neuroprotective effects, can modulate the epigenetic landscape associated with PD. Importantly, we focused on how these interventions impact DNA methylation, histone modifications, and non‐coding RNA regulation, as well as how they interact with key molecular targets such as BDNF, Nrf2, and PGC‐1α. The collective findings highlight a recurring theme: exercise has the capacity to induce widespread epigenetic remodeling in both animal models and humans with PD. DNA methylation changes following aerobic or resistance training are particularly evident in genes involved in synaptic function, inflammation, and mitochondrial health. Parallel changes in histone acetylation suggest that exercise fosters a more transcriptionally permissive chromatin environment, which may underlie its sustained cognitive and motor benefits in PD. Similarly, ncRNAs, especially miRNAs and lncRNAs, emerge as important epigenetic regulators modulated by physical activity. These small molecules help shape post‐transcriptional gene expression in pathways tied to neuronal survival and repair. Although the body of literature on exercise‐induced ncRNA regulation in PD is still developing, early data point to their promise as both mechanistic targets and potential biomarkers. When polyphenols are introduced alongside exercise, preliminary findings suggest the potential for complementary or even synergistic epigenetic effects. Some studies reported enhanced histone acetylation or more pronounced modulation of BDNF and inflammatory markers, whereas others showed no significant additive impact. This inconsistency highlights a key gap in the literature: few well‐designed studies have directly examined the epigenetic consequences of combined interventions in PD, and most available data are either preclinical or extrapolated from other disease contexts. Given the complexity of PD and the growing understanding of its epigenetic basis, it is clear that interventions targeting these modifiable marks could be valuable tools in disease management. However, the field remains at an early stage. More longitudinal, well‐controlled studies, especially those integrating multi‐omics approaches, are needed to map out the temporal dynamics and dose‐responsiveness of epigenetic changes to exercise and polyphenol intake.

As this is a narrative review, our findings should be interpreted with an understanding of the inherent limitations of this approach. The included studies were selected based on thematic relevance and scientific merit but not through a systematic search process. Future meta‐analyses and systematic reviews with predefined criteria will be essential for confirming and quantifying the observed epigenetic trends. In conclusion, this review underscores the potential of lifestyle‐based interventions to reshape the epigenetic architecture in PD. While the individual benefits of exercise and dietary polyphenols are increasingly recognized, their combined impact on epigenetic regulation warrants further investigation. As the science progresses, this integrative approach could offer a personalized, mechanism‐informed strategy for mitigating the course of PD.

## Author Contributions

**Xuenan Cao:** conceptualization (equal), data curation (equal), investigation (equal), methodology (equal), validation (equal), visualization (equal), writing – original draft (equal), writing – review and editing (equal), supervision (equal with Bita Badehnoosh). **Xiantao Cui:** conceptualization (equal), data curation (equal), investigation (equal), methodology (equal), validation (equal), visualization (equal), writing – original draft (equal), writing – review and editing (equal). **Bita Badehnoosh:** conceptualization (equal), data curation (equal), investigation (equal), methodology (equal), supervision (equal), validation (equal), visualization (equal), writing – original draft (equal), writing – review and editing (equal).

## Ethics Statement

The authors have nothing to report.

## Consent

The authors have nothing to report.

## Conflicts of Interest

The authors declare no conflicts of interest.

## Data Availability

Data sharing not applicable to this article as no datasets were generated or analyzed during the current study.
